# Persimmon (Diospyros kaki) fruit: hidden phytochemicals and health claims

**DOI:** 10.17179/excli2015-159

**Published:** 2015-05-04

**Authors:** Masood Sadiq Butt, M. Tauseef Sultan, Mahwish Aziz, Ambreen Naz, Waqas Ahmed, Naresh Kumar, Muhammad Imran

**Affiliations:** 1 National Institute of Food Science & and Technology, University of Agriculture, Faisalabad, Pakistan; 2 Department of Food Sciences, Bahauddin Zakariya University, Multan, Pakistan; 3 Lahore College for Women University, Lahore, Pakistan; 4 Institute of Chemistry, University of São Paulo, São Paulo, Brazil; 5 Department of Chemistry, University of Azad Jammu & Kashmir Muzaffarabad, Pakistan

**Keywords:** persimmon, phytochemicals, astringency, tannins, cardiovascular disorders, oxidative stress, diabetes mellitus

## Abstract

Currently, nutrition and health linkages focused on emerging strategy of diet based regimen to combat various physiological threats including cardiovascular disorders, oxidative stress, diabetes mellitus, etc. In this context, consumption of fruits and vegetables is gaining considerable importance as safeguard to maintain human health. Likewise, their phytochemicals and bioactive molecules are also becoming popular as promising demulcent against various ailments. The current review is an effort to sum up information regarding persimmon fruit with special reference to its phytochemistry and associated health claims. Accordingly, the role of its certain bioactive molecules like proanthocyanidin, carotenoids, tannins, ﬂavonoids, anthocyanidin, catechin, etc. is highlighted. Owing to rich phytochemistry, persimmon and its products are considered effective in mitigating oxidative damage induced by reactive oxygen species (ROS). The antioxidant potential is too responsible for anti-malignant and anti-melanogenic perspectives of persimmon functional ingredients. Additionally, they are effectual in soothing lifestyle related disparities e.g. cardiovascular disorders and diabetes mellitus. There are proven facts that pharmacological application of persimmon or its functional ingredients like proanthocyanidin may helps against hyperlipidemia and hyperglycemia. Nevertheless, astringent taste and diospyrobezoars formation are creating lacuna to prop up its vitality. In toto, persimmon and its components hold potential as one of effective modules in diet based therapy; however, integrated research and meta-analysis are still required to enhance meticulousness.

## Introduction

Fruits and vegetables are important component of human diet and play important role in maintain the human health. The health promoting potentials associated with their consumption are mainly due to presence of bioactive components and these phytochemicals are distinct bioactive molecules widely acknowledged for their beneficial roles in human physiology (Manach et al., 2004[83]). Number of plants gained popularity as wholesome food entities but still many horizons demand researchers attention. Amongst, persimmon (*Diospyros kaki* L.) is one of these nutritious fruits bestowed with strong antioxidant activity (Jung et al., 2005[59]; Igual et al., 2008[50]). 

Persimmon is fleshy fibrous tropical, deciduous fruit belonging to *Ebenaceae* family. It is commonly cultivated in warm regions of the world including China, Korea, Japan, Brazil, Turkey, and Italy (Itamura et al., 2005[54]; Yokozawa et al., 2007[128]). In 2007, the global production of persimmon reached over 3.3 million tons, with 70.0 % from China, 10.0 % from Korea and 7.0 % from Japan. The persimmon is not so popular in European communities but its demand is increasing owing to consumer's awareness regarding its hidden health promoting potential. Mediterranean region is also suitable for persimmon production that has reached up to 110,000 tons (Jung et al., 2005[59]; Luo, 2007[82]; Bubba et al., 2009[18]).

Generally, over 400 species of persimmon are planted globally. Among these, *Diospyros kaki, Diospyros virginiana, Diospyros oleifera,* and *Diospyros lotus* (Bibi et al., 2007[16]) are of significant importance. It is interesting for the readers that *D. kaki* (Japanese persimmon) is the most promising specie (Rahman et al., 2002[105]; Zheng et al., 2006[133]). The popular varieties grown in Japan and their respective characteristics are discussed in Table 1(Tab. 1) (Reference in Table 1: Suzuki et al., 2005[118]).

In some Asian cultures, consumers are aware about the health claims related to persimmon and its functional ingredients. The rich phytochemistry of persimmon opened new avenues of research in diet based regimen to cure various ailments. The health promoting potential of persimmon includes its effectiveness against free-radical production, hypercholesterolemia, diabetes mellitus, cancer, dermal disorders, hypertension, etc. This review is an attempt to elucidate the phytochemistry of persimmon and importance of its bioactive molecules in curing various health disparities. 

## Botanical History and Composition

Being climacteric, persimmon ripening is regulated by ethylene. During climacteric phase, rapid softening occurs resulting jelly-like flesh thus renders persimmon unmarketable within couple of days. Unlike other climacterics, persimmon crop harvested at commercial maturity, produces less ethylene production (below 1.0 nl g^-1^ h^-1^ even at peak production), than that harvested at early mature stage releases higher amounts of ethylene i.e. above 50 nl g^-1^ h^-1^ (Nakano et al., 2002[95]; Harima et al., 2003[46]). The eating quality of persimmon is considered best at the end of the pre-climacteric stage owing to presence of maximum sugars and desired orange color. It is for further info that fruit color is developed just before onset of the ethylene induced respiration (Zheng et al., 2005[134]; Igual et al., 2008[50]; Arnal, et al., 2008[7]). The botanical classification of persimmon fruits is described in Table 2(Tab. 2).

On the basis of extent of astringency, persimmon is divided into astringent (Yamada et al., 2002[127]; Luo, 2007[82]) and non-astringent types (Asgar et al., 2003[8]; Harima et al., 2003[46]; Suzuki et al., 2005[118]). Characteristics of commercially popular cultivars are already been described in Table 1(Tab. 1) (Reference in Table 1: Suzuki et al., 2005[118]). Persimmon is prominent for its nutrition (Achiwa et al., 1997[1]) comprising 80.3 % water, 0.58 % protein, 0.19 % total lipids, 18.6 % total carbohydrates and some minerals (magnesium, iron, zinc, copper, manganese, etc.) and up to 1.48 g and 7.5 mg total dietary fiber, and ascorbic acid respectively (Ozen et al., 2004[98]; Ercisli et al., 2007[31]). Some of the research studies showed that persimmon also contributes in calcium and potassium availability. Sugar content (12.5 g/100 g) are higher in persimmon than other extensively consumed fruits such as apple, peach, pear and orange (Piretti, 1991[102]). Among sugars, sucrose and its monomers (glucose & fructose) are bountiful (Zheng and Sugiura, 1990[132]; Ittah, 1993[55]). The nutritional composition of persimmon fruits (raw and dried) is elucidated in Table 3(Tab. 3).

## Phytochemicals Profile

The intake of phytochemicals is dependent on the consumption of fruits, vegetables, tea etc. (Xing et al., 2001[126]; Miller and Snyder, 2012[92]). These food commodities provide shield against various physiological threats due to the presence of antioxidants i.e. polyphenol, carotenoids, tocopherols (Sakanaka et al., 2005[113]). However, it is important to establish the scientific rationale to defend their use in food chain, as potential nutritionally active ingredients (Dillard and German, 2000[29]). The phytochemicals present in persimmon and their importance is described herein. 

**Figure 1:**
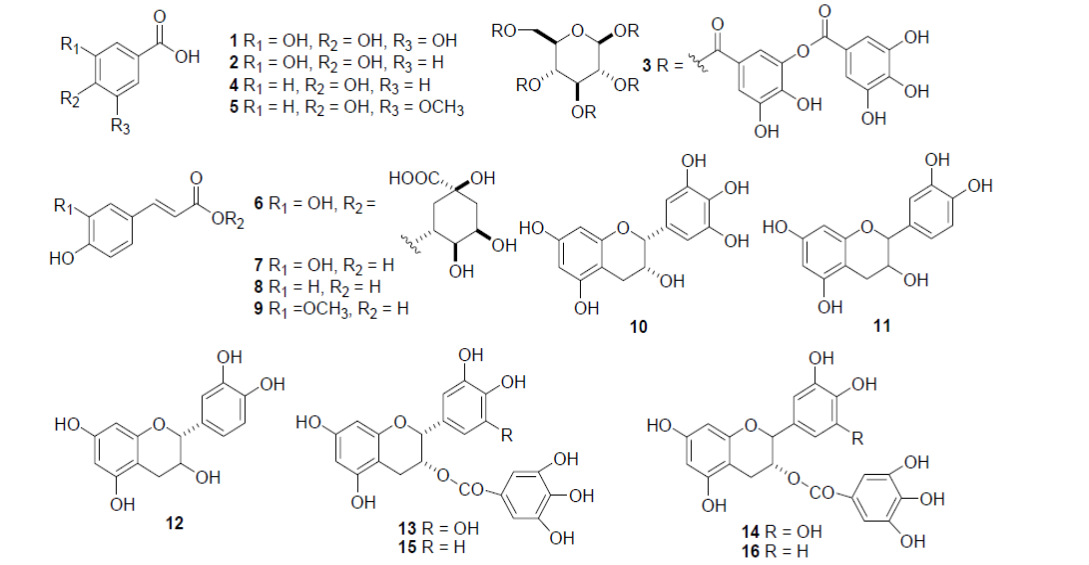
Chemical structures of phenolic acids (1~9) and catechin (10~16). 1: gallic acid, 2: protocatechuic acid, 3: tannic acid, 4: *p*-hydroxylbenzoic acid, 5: vanillic acid, 6: chlorogenic acid, 7: caffeic acid, 8: *p*-coumaric acid, 9: ferulic acid, 10: epigallocatechin, 11: catechin, 12: epicatechin, 13: epigallocatechin gallate, 14: gallocatechin gallate, 15: epicatechin gallate, 16: catechin gallate (Courtesy: Lee et al., 2012)

In persimmon leaves and fruits, some particular components are prevalent e.g. proanthocyanidins (Jung et al., 2005[59]; Suzuki et al., 2005[118]), ﬂavonoid oligomers, tannins, phenolic acids and catechin etc. (Lee et al., 2012[76]; Jo et al., 2003[57]). Carotenoids and tannins (Homnava et al., 1990[49]; Yokozawa et al., 2007[128]) are significant fractions (Figure 1(Fig. 1); Reference in Figure 1: Lee et al., 2012[76]). Nonetheless, dry persimmon residue comprises of 0.16-0.25 g / 100 g polyphenols, 0.002g / 100 g carotenoids and 0.64-1.3 g/ 100 g proteins (Jung et al., 2005[59]). Earlier, Gorinstein et al. (2001[40]) examined the major phenolic contents of persimmon i.e. epicatechin, ferulic acid, gallic acid, protocatechuic acid, vanillic acid, and p-coumaric acid. The beneficial effect of dried persimmon leaves might be due to the presence of phenolic compounds (1.15 g/100g) and fiber (63.48 g/ 100g) contents (Lee et al., 2006[77]).

### Comparison of chemical analysis of fresh and processed persimmon fruit

Fresh and dried persimmons are important nutritional product, which have high content of sugars, as glucose and fructose as a source of energy. The important parameters for determination the quality of fresh and dried persimmons are content of total dry matter and moisture content respectively. It is worth to mention that, the total solids have importance in the dehydration process. Most of the dry matter goes to simple sugars, glucose and fructose, as the most represented in persimmon fruits. According to Fennema (1977[32]), ascorbic acid is considered as an index of nutrient quality during processing and storage of foods, and that's why we examined the content of vitamin C in fresh, frozen-fresh and in all variant of pre-treated dried persimmon sheets. The content of vitamin C in fresh persimmon fruit was in the range on 85.63 - 102.47 mg / 100 g or 460.872 - 541.24 mg / 100 g on dry weight basis. In fresh-frozen persimmon fruit the content of vitamin C was in the range on 103.78 - 112.68 mg / 100 g or 509.225 - 545.137 mg / 100 g on dry weight basis. Generally, persimmon fruit is a good source of ascorbic acid (vitamin C) and also for carotene (pro-vitamin A) sugar, crude fiber and minerals, especially potassium. The contents of all analyzed parameters were higher in fresh-frozen fruit compared to fresh persimmon fruits when the mostly of analyzed parameters were found that had higher content in dried persimmon fruit sheets that were processed from fresh-frozen fruit, the content of glucose and fructose mostly increased in persimmon fruit sheets pre-treated with K_2_S_2_O_5_ where the results were in the range 9.93 - 10.28 % and 8.61 - 8.91 % respectively.

### Major types of phytochemical in Persimmons Fruit

#### Carotenoids

Carotenoids are pigmented compounds abundant in fruits and vegetables have yellow, orange and red color. They usually exist as α, β and γ-forms with specific biological activities. Persimmon is rich in carotenoids especially β-carotenes that can be converted to β-cryptoxanthin. Both these component possesses substantial biological activities (Sarkar et al., 1995[114]; Kumazawa et al., 2002[72]). Various scientists (Sakanaka et al., 2005[113]; Veberic et al., 2010[120]) already reported that β-carotenes are predominant in the persimmon fruit followed by β-cryptoxanthin and α-carotenes. 

#### Tannins

**Figure 2:**
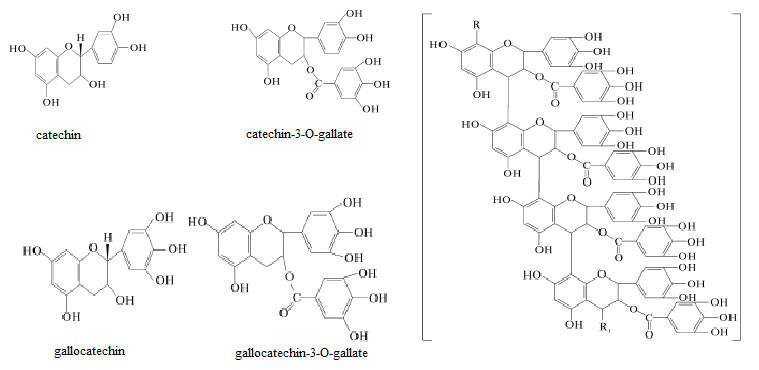
Chemical structures of persimmon tannin and related catechin (Courtesy: Matsuo and Itoo, 1978; Ozen et al., 2004; Gu et al., 2008)

Tannins are one of the important categories of bioactive molecules present in the persimmon meat (Ahn et al., 2002[2]) with molecular weight up to 1.12 × 10^4^da (Bae et al., 2000[10]; Wu and Hwang, 2002[124]; Jo et al., 2003[57]). They contain gallic acid residues linked with glucose *via* glycosidic bonds. Considering the chemical properties, tannins can be split up into two broader groups i.e. hydrolyzable and non-hydrolyzable. However, on the basis of structural configurations, there are three major categories i.e. complex, condensed, and hydrolyzable tannins. Regarding astringency, about 2 % of condensed soluble tannins are observed in particular varieties due to the formation of salivary protein complexes (Manach et al., 2004[83]; Bibi et al., 2007[16]; Yokozawa et al., 2007[128]; Gu et al., 2008[43]). On eating an astringent persimmon, the tannin cells present in the fruit are crushed and soluble tannins are released thus resulting astringent sensation (Asgar et al., 2003[8]). However, this hedonic lacuna can be masked through controlled reaction with protein. Conversion of soluble tannins into insoluble form results in disappearance of astringency (Igual et al., 2008[50]). Additionally, flavano-ellagitannin (molecule of flavan-3-ol coupled with hydrolyzable tannin moiety through carbon-carbon linkage) and procyanidino-ellagitannin (proanthocyanidins and ellagitannins) are also substantial phytochemical moieties present in persimmon. Previously, some scientists attempted to identify the degraded tannin products. For the purpose, persimmon tannins were deposited with K_2_HPO_4_ and reactions were initiated using benzyl mercaptan. Their results indicated that there are four important components i.e. gallo-catechin, catechin, catechin-gallate and gallocatechin-gallate. The structures of these components are portrayed in Figure 2(Fig. 2) (References in Figure 2: Matsuo and Itoo, 1978[89]; Ozen et al., 2004[98]; Gu et al., 2008[43]).

#### Phenolic compounds

**Figure 3:**
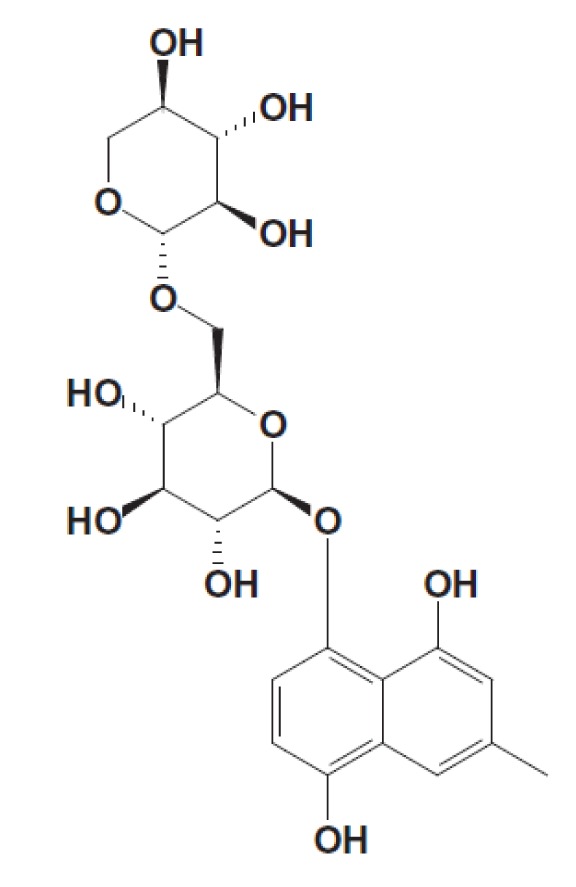
Novel phenolic compound 4,8-dihydroxy-6-methyl-1-naphthalenyl 6-O-β-D xylopyranosyl-β-D glucopyranoside was isolated from Diospyros kaki. (Courtesy: Gondo et al., 1999)

The phenolics present in persimmon are further categorized into water soluble/ extractable [EPP] and water insoluble/non-extractable [NEPP]. The absorption patterns of both these categories differ from each other as EPPs are directly absorbed from the digestive tract. In contrary, NEPPs are not absorbed in the intestine and wasted in feces (Barry et al., 1986[12]; Bravo et al., 1992[17]). The total polyphenols are observed to be 1.45 mg / 100 g whilst gallic acid contents 190.2-252.2 µg / 100 g in fresh persimmon (Sakanaka et al., 2005[113]). Recently, it has been observed that predominant polyphenols in fresh leaves are water-soluble, and the contents reached a maximum (2.40 % w/w) in June, afterwards gradually decreases (Kawakami et al., 2010[62]). The flavonoid subclass of phenols includes minor flavonoids (flavanones and dihydroflavonols), proanthocyanidins, flavones and flavonols. Some of these flavonoids are present in persimmon such as proanthocyanidins and flavanols (Yokozawa et al., 2007[128]). Moreover, anthocyanidins are pigmented aglycones of anthocyanins usually water-soluble including pelargonidin, cyanidin, paeonidin, delphinidin, glucopyranosides, petunidin, and malvidin (Gondo et al., 1999[37]; Ikegami et al., 2009[51]; Dembitsky et al., 2011[26]). Likewise, a new phenolic metabolite, 4, 8-dihydroxy-6-methyl-1-naphthalenyl 6-O-β-D xylopyranosyl- β-D glucopyranoside (Figure 3(Fig. 3)) was also extracted from persimmon (Gondo et al., 1999[37]).

#### Proanthocyanidins (PAs)

**Figure 4:**
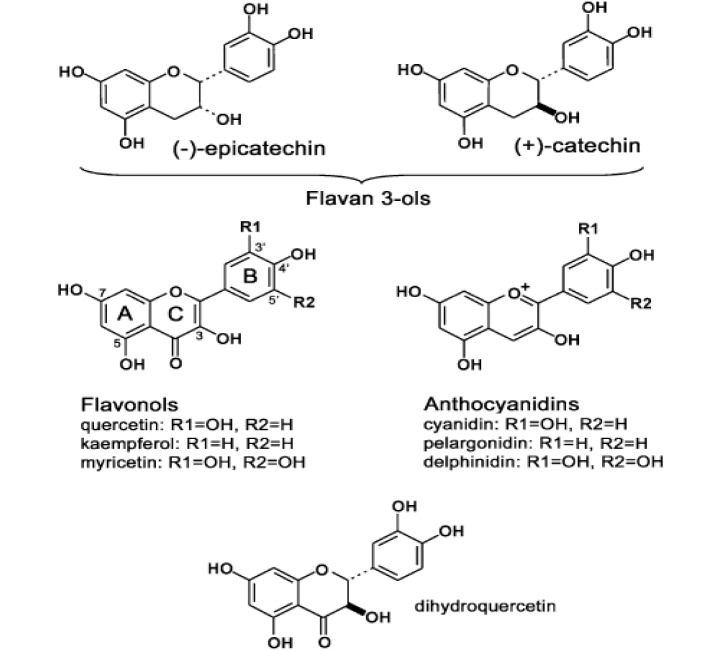
Chemical structures of Xavonoid aglycones relevant to this study. Flavan-3-ols are monomers of proanthocyanidins (PAs), whereas anthocyanidins are aglycones of anthocyanins, which contain sugar moieties attached to various hydroxyl groups at A, B, and C rings (Courtesy: Ikegami et al., 2009)

These components accumulate in enormous amount in persimmon fruit during early development stages. They are secondary metabolites providing safeguard against various problems including environmental stress (Akagi et al., 2010[3]). Biochemically, they are colorless polymers (Plumb et al., 1998[103]; Yokozawa et al., 2007[128]) formed after condensation of ﬂavan 3-ol units (Dixon et al., 2005[30]). Ikegami et al. (2009[51]) identified the catalytic and regulatory mechanisms of phenylpropanoid metabolism and elaborated that PAs usually consisting of Xavan-3-ol units are formed that also include the bioactive moieties like xavonols and glycosylated anthocyanidins (Figure 4(Fig. 4); Reference in Figure 4: Ikegami et al., 2009[51]). They comprised of two to many units of catechin with molecular weight of 1.38 × 10^4^ Da (Ikegami et al., 2007[52]). PAs are present in higher amounts in astringent (A)-type fruits even after they reached full maturity. In comparison, non-astringent (NA)-type fruits lose these functional ingredients before maturation (Ikegami et al., 2009[51]). In recent years, more attention has been paid to PAs and their monomers because of associated health claims. They are also effective in providing bitter mouth feel & color and of course effect on consumption too (Lee et al., 2008[79]; Zhao et al., 2007[131]).

#### Catechins

These belong to group of bioactive molecules that provide protection against various ailments (Suzuki et al., 2005[118]). They are mostly present in grapes, bananas, berries, cocoa and green tea (Khokhar and Magnusdotir, 2002[65]). In persimmon, soluble tannins consist of catechin (Yokozawa et al., 2007[128]), catechin-3-gallat, galocatechin and galocatechin-3-gallat (Akyidiz et al., 2004[5]). They further contain substantial amounts of gallic acid esters, for instance, epicatechin gallate and epigallocatechin gallate (Wu and Hwang, 2002[124]; Suzuki et al., 2005[118]; Gu et al., 2008[43]). The catechins contents are higher in astringent persimmons than that of non-astringent cultivars (Suzuki et al., 2005[118]). 

Oleanolic acid (OA) and ursolic acid (UA) are also important bioactive components of persimmon that varied from traces to 88.57 and traces and 27.64 μg/g FW, respectively (Zhou et al., 2010[135]). Furthermore, dietary fiber is present approximately 1.20-1.76 % whilst soluble fiber accounts for 0.52-0.92 %. The heating/blanching of persimmon's peels at 50 °C can yield better quality dietary fiber powder and resultant product holds several food applications for the preparation of fiber-fortified foods (Akter et al., 2010[4]). In a research study, Chen et al. (1999[22]) isolated novel 18, 19-secoursane triterpenoids, kakisaponin B (_1_) and kakisaponin C (_2_), an ursane type 28-nortriterpene, kakidiol and known triterpenoid rosamultin from the leaves of *Diospyros kaki.* It is interesting to express that persimmon peel contains large amount of polyphenolic compounds that are necessary to protect the inner fleshy mass (Kim et al., 2006)[68].

## Health Claims

Persimmon leaves have beneficial effects against oxidative stress, hypertension, diabetes mellitus and its complications, and atherosclerosis (Kotani et al., 2000[70]; Wang et al., 2004[121]). The bioactive components present in it especially carotenoids and tannin are helpful in quenching free radicals, decreasing cardiovascular risk factors (blood pressure & cholesterol), and reducing the risks of diabetes mellitus along with effectiveness against cancer insurgence (Park et al., 2002[99]; Lee et al., 2006[77]). 

The tannins present in persimmon are eventually responsible for curing physiological threats. They possess antibacterial (Kawase et al., 2003[63]), anti-allergic (Kotani et al., 2000[70]), free radicals scavenging (Sakanaka et al., 2005[113]), lowering blood pressure (Jo et al., 2003[57]), anticancer and antioxidant activities (Gali et al., 1992[34]). The antioxidant activities of tannin are due to presence of nucleophiles moieties (Laranjinha et al., 1994[74]; Ahn et al., 2002[2]) along with some antimutagenic properties (Achiwa et al., 1997[1]) through inhibition of nitrogen reactive compounds. They are also effective in reducing the incidence of stroke (Wu and Hwang, 2002[124]; Jung et al., 2005[59]) and hypertensive disorders (Sakanaka et al., 2005[113]). Similarly, flavonoids obstruct the activity of angiotensin-converting enzyme that elevates the blood pressure, and inhibit cyclooxygenase that forms prostaglandins. Some *in vitro* studies have depicted inhibitory actions of flavonoids in prevention of platelet aggregation, and thrombosis formation. According to survey conducted in USA, inverse relationship exists between persimmon consumption and coronary heart disease (Hertog et al., 1995[47]; Lee et al., 2006[77]). The carotenoids and catechins also hold anticancer perspectives against various cancer cell lines (Achiwa et al., 1997[1]; Prakash et al., 2000[104]). Anthocyanidins resembles to other flavonoids as execute antioxidative activities in vivo and in vitro (Rice-Evans et al., 1997[108]) as well as anti-mutagenic properties in vivo. Additionally, persimmon fruit possesses hypocholesterolemic and antioxidant potential (Yokozawa et al., 2007[128]; Gu et al., 2008[43]; Sun et al., 2011[117]; Gato et al., 2013[35]). The detailed health claims associated with persimmon are described herein and some highlights are also being placed in Table 4(Tab. 4) (References in Table 4: Carotenoids: Mathews-Roth, 1993[85]; Gaziano and Hennekens, 1993[36]; Tannin: Wu and Hwang, 2002[124]; Kotani et al., 2000[70]; Sakanaka et al., 2005[113]; Gali et al., 1992[34]; Achiwa et al., 1997[1]; Flavanols: Gondo et al., 1999[37]; Suzuki et al., 2005[118]; Rice-Evans et al., 1997[108]; Anthocyanidins: Han et al., 2002[44]; Jung et al., 2005[59]; Yokozawa et al., 2007[128]; Gorinstein et al., 1998[38]; Gu et al., 2008[43]). 

### Coronary Care

In the developed societies, coronary heart diseases (CHD) are major reason for human morbidity and mortality. Several researchers attempted to identify major risk factors for cardiovascular health ailments. In this regard, accumulated evidences suggested that atherosclerosis, imbalance lipid profile, hypertension, and high blood pressure are major causative agents. Moreover, apolipoproteins A-I, B, CI and CIII are also important determinants (Larsson et al., 2013[75]; Antman and Jessup, 2014[6]). Moreover, free radicals can initiate the oxidation of low density lipoproteins (LDL) is another risk factor responsible for onset of atherosclerosis (Aviram, 1993[9]; Chen et al., 2003[21]; Matsuura et al., 2006[90]). The oxidation of LDL is closely linked with accumulations of lipids in the arteries thus enhancing the risk of atherosclerosis (Gorinstein et al., 2000[39]; Mashima et al., 2001[84]; Chen et al., 2003[21]). In the last couple of decades, nutritionists and dietitians focused on development of such dietary strategies that include the utilization of antioxidants rich sources in order to reduce the risk of cardiovascular disorders. Therefore, diets rich in these natural antioxidants are in demand (Kromhout et al., 2002[71]; Park et al., 2006[100]).

The consumption of higher amounts of fruits and vegetables is responsible for are is associated with lower concentrations of total and low-density lipoprotein cholesterol and with the risk of CVD per se in a dose-response manner (Hertog et al., 1995[47]; Dauchet et al., 2009[25]; Baldrick et al., 2011[11]; Ros et al., 2013[111]; Woodside et al., 2013[123]). Persimmon is one of nutritious entities that hold hypocholesterolemic effects. The reasons include presence of bioactive compounds that possess the plasma lipid lowering and antioxidant properties (Gorinstein et al., 2000[39]; Kim et al., 2009[69]). The bioactivity of persimmon is associated with its water-soluble dietary ﬁbers, minerals, trace elements and phenolics (Hertog et al., 1995[47]). Dietary fiber is present in persimmon in the amounts of 1.20-1.76 % and soluble fiber accounts for 0.52-0.92 %. Dietary fiber holds well defined effects on the lipid metabolism thus persimmon can help in meeting the recommended dietary allowance of dietary fiber i.e. 30-45 g (Roller et al., 2007[110]). Along with dietary fibers, minerals and essential trace elements present in persimmon fruits can prevent coronary atherosclerosis and its complications (Baxter et al., 1996[14]; Kiechl et al., 1997[66]). 

Supplementation of persimmon can improve the plasma lipid metabolism and total antioxidant activity (Gorinstein et al., 1998[38]). Number of scientific investigations provided evidences that the whole persimmon or its parts hold lipid lowering effects in hypercholesterolemic rats. Persimmon leaf powder improved plasma and hepatic lipid levels profile partly via increased fecal lipids excretion (Jung et al., 2012[60]). These beneficial effects may be due to the properties of its phenolic compounds (1.15 g / 100 g) and high fiber (63.48 g / 100 g) content (Innami et al., 1998[53]; Gorinstein et al., 1999[38]). In another study, Lee et al. (2006[77]) supplementation powdered whole persimmon leaf (5 %) and observed that supplementation results in lowering of blood cholesterol and triglyceride, while increased ratio of HDL-C/total-C was also observed. In another study, Matsumoto et al. (2006[86]) supplemented diet with young persimmon fruit (10 %) that reslted in similar results i.e. lowering of total & LDL cholesterol and triglyceride. They reported that the improved lipid profile might be due to increased expression of cholesterol 7 alpha-hydroxylase (CYP7A1) gene's expression. CYP7A1 regulates bile acid synthesis thus holds imperative role in balancing cholesterol homeostasis. As mentioned earlier, higher amounts of LDL are also harmful for human health due to their sucesptibility to oxidation that can further lead to an inflammatory burst resulting in proliferation of endothelial vascular cell to arteries thus leading to the menace of atherosclerosis (Singh et al., 2007[115]). The persimmon and its bioactive molecules like flavone can inhibit proliferation of rat vascular smooth muscle cells exposed to high levels of LDL. They may exert vascular protection by inhibiting vascular smooth muscle cell growth associated with hypercholesterolemia (Ouyang et al., 2004[97]). Later, Matsumoto et al. (2008[88]) conducted studies to check the influence of young persimmon fruit (YP) at 5 % for 10 weeks. YP significantly lowered plasma chylomicron, very low-density lipoprotein (VLDL) and LDL cholesterols, and triglyceride. Additionally, the lipid lowering response of dietary persimmon (7.0 %) was accompanied by an elevation of fecal bile acid excretion and eventually led the hepatic cholesterol and triglyceride values to become normal (Lee et al., 2006[77]). The persimmon-vinegar is traditional fermented product and is used in traditional medicines and cuisine application. However, persimmon-vinegar (2 mL / kg of body weight) holds the potential to significantly decrease the serum triglyceride & cholesterol along with reducing the liver cholesterol (Moon and Cha, 2008[93]). 

In some Asian countries, persimmon leaves are used in tea formulations to cure hypertension. The clinical trials revealed that Renin angiotensin system (RAS) and angiotensin converting enzyme (ACE) are important targets to control or manage the hypertension. Sa et al. (2005[112]) separated the anticoagulant fraction from leaves of persimmon. They reported that extraction fraction delayed thrombin time (TT), activated partial thromboplastin time (APTT), and prothrombin time (PT). The effects could be due to presence of astragalin that has been reported to suppress ACE activity (Paul et al., 2006[101]; Cho et al., 2014[24]). The importance of ACE inhibitors in the chronic treatment of various cardiovascular diseases is now well established (Remuzzi and Ruggenenti, 2006[107]; Lee et al., 2007[78]) and several ACE inhibitors are in the market. Later, Matsumoto et al. (2010[87]) confirmed hypolipidemic effects & bile acid-binding properties of persimmon. They were of the view that persimmon fruits enhanced the bile acid excretion through feces. The excretion of bile acids is closely linked with reduced concentration of lipids in liver and blood. The mechanism of action remained centered around up-regulation of expression of the sterol regulatory element-binding protein-2 gene, 7α-hydroxylase, and the low-density lipoprotein receptor. The effectiveness of dietary persimmon prevented the incidence of stroke due to the radical scavenging action and inhibition of lipid peroxidation (Ahn et al., 2002[2]). 

In the nutshell, the cholesterol lowering effects of persimmon and its products are due to decreased cholesterol absorption, cholesterol & fatty acids synthesis. Indeed, phenolic compounds and dietary fiber are the main constituents responsible for its cardioprotective effects.

### Antioxidant potential and protection against DNA damage

The process of oxidation is essential in vitality of life coupled with production of free radicals. A natural balance exists between the production of reactive oxygen species (ROS) and the endogenous antioxidant defense system. These reactive free radicals results in induction of oxidative stress that can damage the cell bound structures and some essential components e.g. proteins and DNA. However, the imbalance can be overcome through supplementation of antioxidants. In this regard, polyphenols, carotenoids, flavonoids, tocopherols, anthocyanins, and tannins are of considerable importance (Ahn et al., 2002[2]; Lee et al., 2006[77]; Butt et al., 2008[19]; Gu et al., 2008[43]). 

Persimmon is naturally bestowed with bioactive molecules including phenolic compounds such as proanthocyanidins, tannins, catechins (Suzuki et al., 2005[118]), carotenoids, sinapine, leucoanthocyanidin, catechin, kaempferol, quercetin, etc. (Park et al., 2006[100]). Most of these bioactive components scavenge free radicals, bind metals, and inhibit the lipid peroxidation. The condensed tannins and ﬂavonoids also contributes towards antioxidant potential of persimmon thus can be considered as effective agents to prevent various lifestyle related disorders (Gali et al., 1992[34]; Gorinstein et al., 1994[42]; Lee et al., 2006[77]). Persimmon also contains carotenoids, polyphenols, and ascorbic acid that have antioxidant properties (Yokozawa et al., 2007[128]) and most of them can play protective roles against oxidative stress-related diseases (Homnava et al., 1990[49]; Suzuki et al., 2005[118]; Bubba et al., 2009[18]). Proanthocyanidins (PAs) present in persimmon are also considered as potent antioxidants thus can be an important factor for reducing oxidative stress induced ailments. However, their efficacy for medicinal purposes needs further elaborations. In one study, oligomer and polymeric PAs suppressed oxidative stress involving the mechanism like scavenging free radicals (oxygen and nitrogen reactive species) and lipid peroxidation inhibition (Kim and Yokozawa, 2009[69]). Additionally, PAs had a strong inhibitory effect on the murine tyrosinase and melanin synthesis that could be again due to suppression of oxidative stress (Kim et al., 2010[67]). 

The scientists tested the antioxidant and persimmon and it bioactive through various *in vitro* and *in vivo* assays. In one such study, Chen et al. (2008[23]) observed the radical scavenging activities against ABTS and DPPH radicals of the Mopan persimmon i..e 23.575 and 22.597 microm trolox eq/g f.w., respectively. The proanthocyanidin supports the protective potential oxidative damage under aging process (Lee et al., 2008[79]). Later, Lee et al. (201[80]) studied and reported t he positive aspects of the oral administration of PAs in spatial and object recognition impairment in Senescence-accelerated mouse prone/8 (SAMP8). The positive impact of persimmon in providing protection against memory impairment with aging is due to presence of oligomeric proanthocyanidins (Yokozawa et al., 2014[129]). Previously, it was reported that phytochemical rich fractions (acetone-extract) suppresses the tyrosinase expression thus inhibiting melanin biosynthesis in mouse B16 melanoma cells (Ohguchi et al., 2010[96]). Consequently, extracts from persimmon leaves can be considered important in natural skin care owing to their antioxidant perspectives (Mure et al., 2007[94]). 

### Persimmon preventive role against cancer insurgence and DNA damage 

Several researchers across the globe attempted to provide evidences regarding anticancer perspectives of persimmon and its bioactive components (Jung et al., 2013[58]). In this context, Khanal et al. (2010[64]) studied the anticancer properties of 24-hydroxyursolic acid. They reported that selected compound inhibited cell proliferation through activation of AMP-activated protein kinase (AMPK) pathway in colon cancer (HT-29 cells) along with inhibiting cyclooxygenase (COX-2) expression, inducing apoptosis by activation of poly (ADP-ribose) polymerase (PARP), caspase-3, and phosphorylation of p53 at Ser15. In addition, 24-hydroxyursolic acid blocked the EGF-induced extracellular signal-regulated kinase (ERK) phosphorylation. Recently, Kim et al. (2010[67]) investigated the effect of an acetone extract of D. kaki leaves (KV-1) on HL-60 cell differentiation that indicated that persimmon leaves has the ability to enhance HL-60 cell differentiation and suggest that it may be useful in blood cancer (acute promyelocytic leukemia) therapy. The mode of action includes inhibition of protein kinase C (PKC) and ERK pathways. The extracts of persimmon prevented H_2_O_2_-induced DNA damage to human peripheral lymphocytes (Kim et al., 2006[68]). Previously, Bei et al. (2005[15]) pretreated NG108-15 cells with Flavonoids from the leaves of *Diospyros kaki* L (FLDK-P70) that resulted in alleviation of H_2_O_2_-induced cell injury and apoptosis by up-regulation Bcl-2 expression and improving redox imbalance. The FLDK-P70 further reported to modulate the decline in the intracellular endogenous antioxidants including glutathione and glutathione peroxidase. 

**Figure 5:**
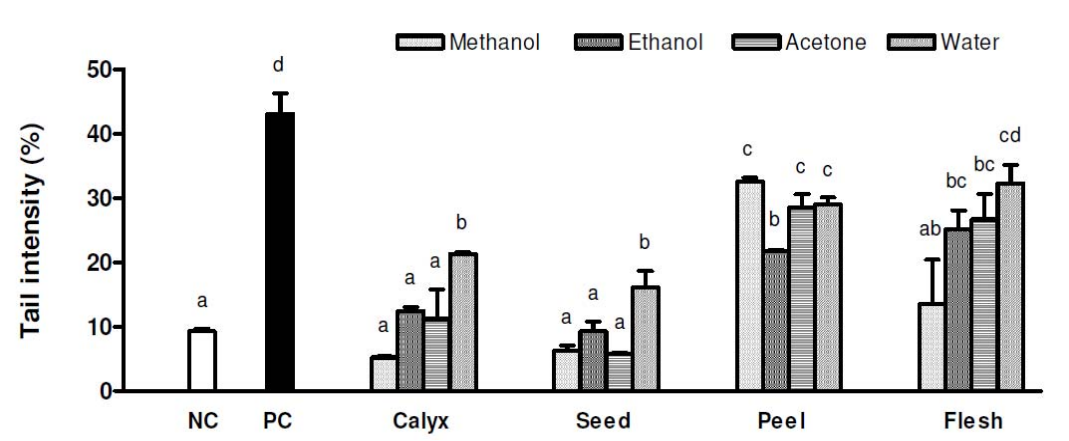
The effect of extracts (50 μg/ml) from persimmon calyx, seed, peel and flesh by methanol, ethanol, acetone and water on 200 μM H2O2-induced DNA damage in human leukocyte. Values are mean with standard deviation of triplicate experiments. NC, 1% DMSO treated negative control; PC, 200 μM H2O2 treated positive control. Values not sharing the same letter are significantly different from one another (p < 0.05) by Duncan's multiple range tests. (Courtesy: Jang et al., 2010)

Reactive oxygen/nitrogen species or free radicals can cause DNA damage that can ultimately leads to cancerous growth and important mediator in aging and degenerative disorders (Michel et al., 2012[91]). Carotenoids and flavonoids are amongst the high potency natural antioxidant with excellent abilities to scavenge free radicals (Hanasaki et al., 1994[45]; Fiedor and Burda, 2014[33]). Moreover, the phytochemicals like polyphenols and anthocyanins are also linked with anticancer perspective of the foods. These bioactive components can reduce the DNA damage caused by various genotoxic factors (Kapiszewska et al., 2005[61]). Researchers attempted to study the effects of persimmon and its fraction and one such study was conducted by In-Cheol et al. (2010). They observed that 50 mg/ml of extracts provided higher protection against H_2_O_2_ induced DNA damage (Figure 5(Fig. 5); Reference in Figure 5: Jang et al., 2010[56]). These effects could be due to presence of carotenoids and flavonoids (Takahashi et al., 2006[119]), tannin (Lee et al., 2007[78]) and ascorbic acid**.**

The antioxidant potential of persimmon can be exploited to cure various maladies including degenerative disorders, cancer, and improving the skin tone. Thus persimmon and its bioactive molecules are potential as food or cosmetic applications. 

### Diabetes mellitus 

Despite the heterogeneity, hyperglycemia and allied complications are the foremost common metabolic disorders in diabetic patients. Diabetes mellitus can be caused by multiple factors that include hyperglycemia, decreased insulin concentrations or its sensitivity. Sometimes, the beta cells of pancreas are damaged thus limiting the insulin production. These conditions results in insulin-stimulated improper glucose transport in the body (Ziel et al., 1988[136]). In order to overcome these problems, some targeted anti-diabetic drugs are in use as glucose-lowering agents including metformin, rosiglitazone and pioglitazone (Yonemitsu et al., 2001[130]). Drug therapies often lose their effectiveness with time and natural compounds are gaining attention for curing diabetes mellitus and allied complications (Wild et al., 2004[122]; Lapshina et al., 2006[73]). In Asian communities, natural products are more popular to cure type 2 diabetes mellitus (Sa et al., 2005[112]). However, these drugs often accompany escalating side effects. In response, natural foods are replacing these drugs to control the menace of diabetes mellitus and its complications (Sultan et al., 2009[116]). 

Persimmon owing to rich phytochemistry can contribute imperative role in prevention and cure of diabetes mellitus. In this regard, Kawakami et al. (2010[62]) studied the effects of persimmon leaf in conjunction with dietary starch and observed dose-dependent decrease in the blood glucose level in rodent modeling studies. They were of the view that inhibition of pancreas alpha-amylase could be one of major mechanisms responsible for the antidiabetic role of persimmon (Ramasubbu et al., 2004[106]). However, the antidiabetic effects are dependent on degree of polymerization of bioactive components of persimmon. Lee et al. (2007[81]) explicated that the polymeric proanthocyanidins inhibited α-amylase significantly but inhibitory potential of oligomers was little weaker against the same enzyme. In contrast, the inhibitory potential of oligomeric proanthocyanidins against α-glucosidase was higher than oligomeric proanthocyanidins. More recently, Xiao et al. (2013[125]) reviewed the role of dietary polyphenols in diabetes mellitus with special reference to their anti α-amylase activities. They presented highlights of different researches and concluded that the molecular structures influence the inhibition e.g. hydroxylation of flavonoids, unsaturated 2,3-bond in conjugation with a 4-carbonyl group, The galloylated catechins have higher inhibition than nongalloylated catechins; the catechol-type catechins were stronger than the pyrogallol-type catechins, glucoside moiety attached with cyanidin results in higher inhibition than galactoside and diglucoside, and ellagitannins with β-galloyl groups are more effective than α-galloyl and nongalloyl ellagitannins. In comparison, glycosylation, methylation and methoxylation of flavonoids results in reduced antidiabetic activity as witnessed from decreased inhibition of α-amylase.

Persimmon leaves may also have a therapeutic potential against diabetes due to modulation of insulin dependent glucose transport. The persimmon leaves influence the insulin-stimulated muscular glucose transport and thus can be used as insulin sensitizers for the treatment of diabetes (Kawakami et al., 2010[62]). The ethyl acetate-extracted fraction, water-extracted and ethanol-precipitated fraction of persimmon leaves have potential value in the treatment of diabetes. The mechanism of action of the antioxidant is related to the hypoglycemic effects of extracts from persimmon leaves (Deng et al., 2012[27]).

Diabetes mellitus results in oxidative stress that involves the due to non-enzymatic glycation of proteins that can lead to the production of undesirable Schiff base and Amadori products (Robertson et al., 2003[109]). In addition, diabetes induced oxidative stress further weakens enzyme mediated antioxidant defense system of the body. Thus, dwindling of oxidative stress is important to cure pathological damage and diabetes complications (Basta et al., 2004[13]; Lapshina et al., 2006[73]). Persimmon is rich in antioxidants and has potent scavenging action against reactive oxygen species (Han et al., 2002[44]; Jung et al., 2005[59]) that can be exploited to control diabetes complications. Lee et al. (2008[79]) investigated the protective effect of proanthocyanidins from persimmon peel in a db/db type 2 diabetes model. The increased oxidative stress in db/db mice was attenuated by the daily administration of 10 mg/kg body weight oligomers. The acetyl-coenzyme A carboxylase mRNA decreased and hepatic carnitine palmitoyltransferase-I mRNA increased as suggested by Moon and Cha (2008[93]). The persimmon polyphenols (PPP) can be categorized into high molecular-persimmon peel polyphenols (HMPPP) and low molecular-persimmon peel polyphenols (LMPPP). The low molecular fractions or LMPPP contains higher amounts of oligomeric proanthocyanidins are more effective than HMPPP (rich in polymeric proanthocyanidins) in reducing the extent of oxidative stress (Yokozawa et al., 2007[128]). Persimmon and its bioactive components/fractions also attenuated the protein expressions of cyclooxygenase-2 and inducible nitric oxide synthase (Yokozawa et al., 2007[128]; Lee et al., 2008[79]). Moreover, persimmon can ameliorate inflammatory responses in diabetes suppression owing to its ability to scavenge free radicals and elevating the ratio of reduced glutathione/oxidized glutathione (Lee et al., 2007[78]). In another investigation, oral administration of persimmon extract at 50 and 100 mg/kg body weight per day to diabetic rats was found to possess significant dose dependant hypoglycemic and hypolipidemic activity (Dewanjee et al., 2009[28]). In the nutshell, administration of persimmon or its functional ingredients may be effective in especially designed preventive strategies for diabetes mellitus or reducing the extent of diabetes complication through lowering blood pressure, blood lipids, and modulating of oxidative stress and inflammatory responses (Lee et al., 2008[79]).

## Conclusions

Persimmon is naturally bestowed with bioactive molecules including proanthocyanidins, ﬂavonoids, tannins, phenolic, carotenoids, dietary fiber, and etc. Persimmon leaves and fruit have imperative significance for coronary health because of hypocholesterolemic, anti-atherosclerosis and antioxidant perspectives. Although hypotensive and anticancer responses have been reported for persimmon and its bioactive especially condensed tannin and flavonoids too but yet demands further probing to unveil their therapeutic mechanisms. In the last, utilization of persimmon and its bioactive components can be effective in reducing the burden of diabetes mellitus. However, coherent and systematic research is still required to bring meticulousness. 

## Figures and Tables

**Table 1:**
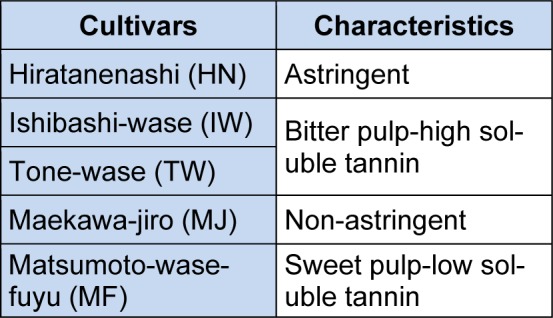
Characteristics of commercially renowned cultivars (Courtesy: Suzuki et al., 2005)

**Table 2:**
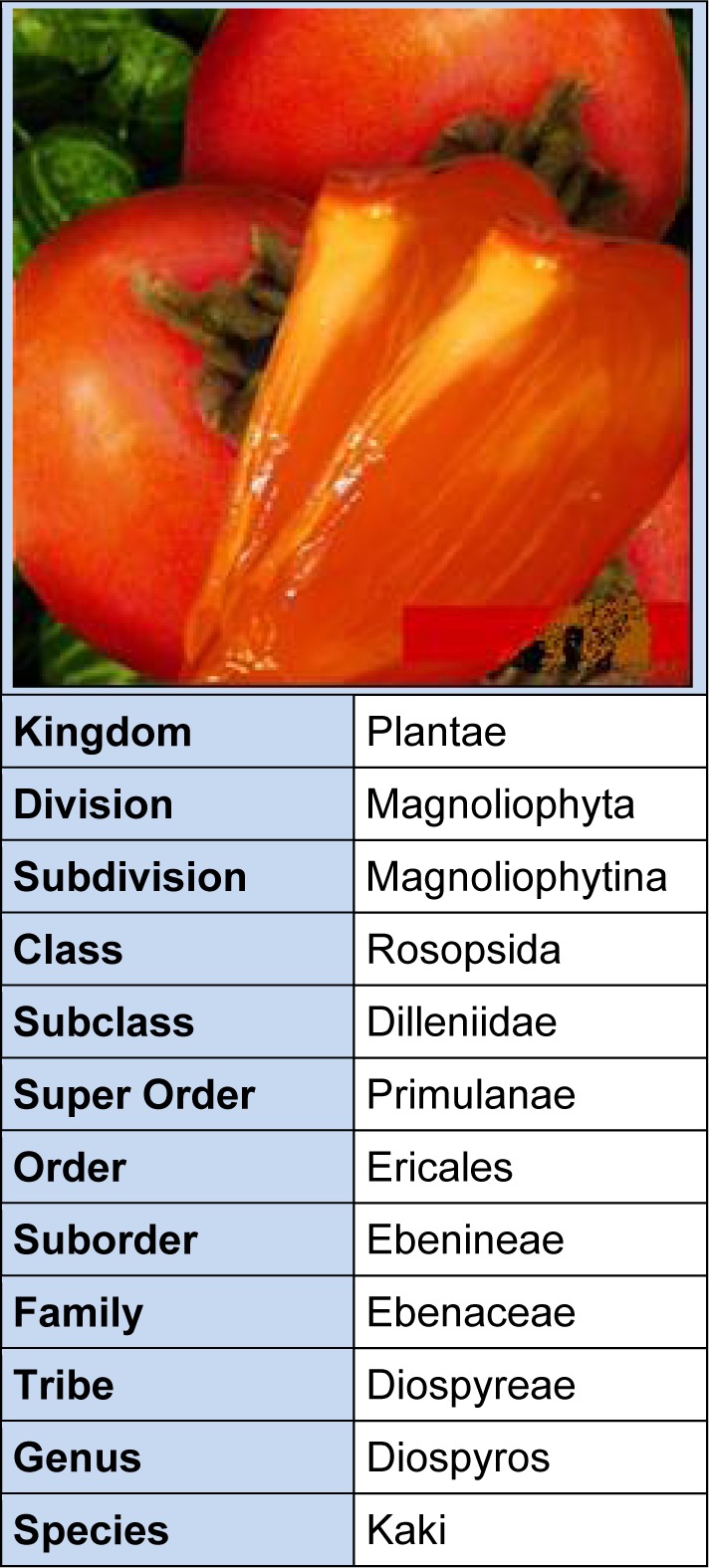
Botanical classification

**Table 3:**
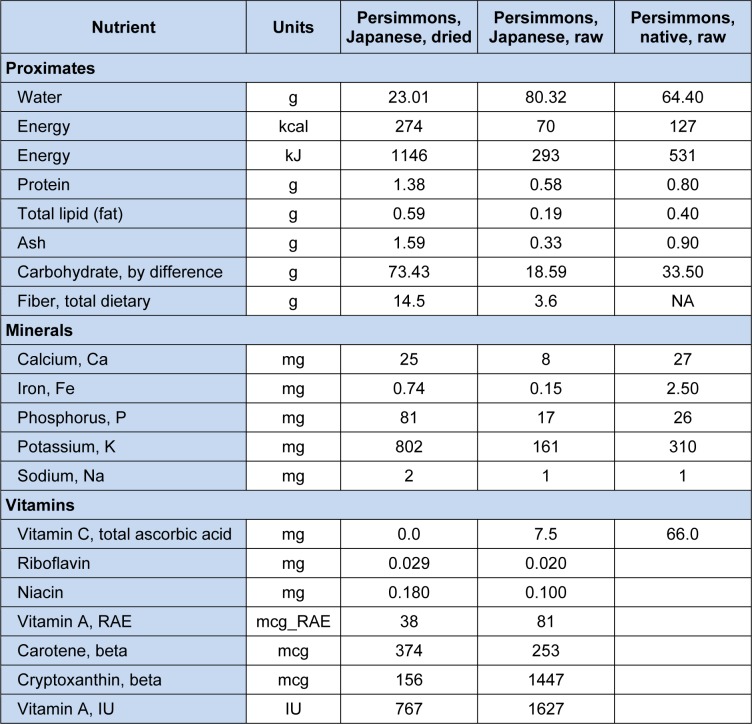
Nutritional value of persimmons (Value per 100 grams)

**Table 4:**
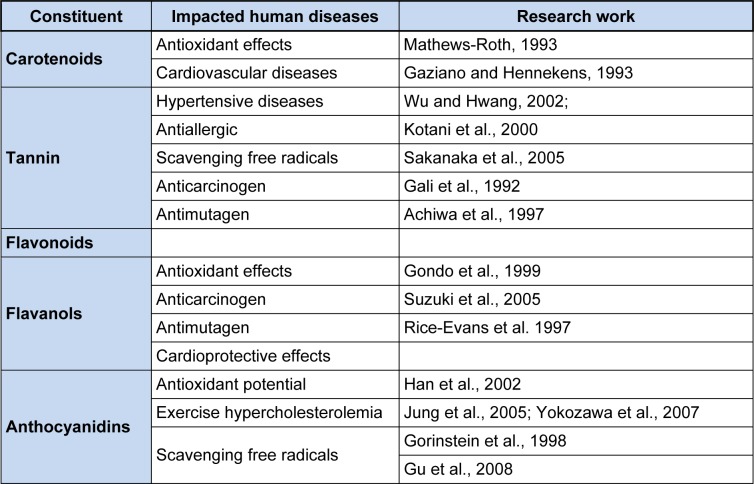
Constituents of persimmon that have a positive impact on human health

## References

[1] Achiwa Y, Hibasami H, Katsuzaki H, Imai K, Komiya T (1997). Inhibitory effects of persimmon (Diospyros kaki) extract and related polyphenol compounds on growth of human lymphoid leukemia cells. Biosci Biotechnol Biochem.

[2] Ahn HS, Jeon TI, Lee JY, Hwang SG, Lim Y, Park DK (2002). Antioxidative activity of persimmon and grape seed extract: in vitro and in vivo. Nutr Res.

[3] Akagi T, Ikegami A, Yonemori K (2010). DkMyb2 wound-induced transcription factor of persimmon (Diospyros kaki Thunb.), contributes to proanthocyanidin regulation. Planta.

[4] Akter MS, Ahmed M, Eun JB (2010). Effect of blanching and drying temperatures on the physicochemical characteristics, dietary fiber composition and antioxidant-related parameters of dried persimmons peel powder. Int J Food Sci Nutr.

[5] Akyıldız A, Aksay S, Benli H, Kıroğlu F, Fenercioğlu H (2004). Determination of changes in some characteristics of persimmon during dehydration at different temperatures. J Food Eng.

[6] Antman EM, Jessup M (2014). Clinical practice guidelines for chronic cardiovascular disorders:a roadmap for the future. JAMA.

[7] Arnal L, Besada C, Navarro P, Salvador A (2008). Effect of controlled atmospheres on maintaining quality of persimmon fruit cv. Rojo Brillante. J Food Sci.

[8] Asgar MA, Yamauchi R, Kato K (2003). Modification of pectin in Japanese persimmon fruit during the sun-drying process. Food Chem.

[9] Aviram, M (1993). Modified form of low density lipoprotein and atherosclerosis. Atherosclerosis.

[10] Bae DK, Choi HJ, Son JH, Park MH, Bae JH, An BJ (2000). The study of developing and stability of functional beverage from korean persimmon (Diospyros kaki L. folium) leaf. Korean J Food Sci Technol.

[11] Baldrick FR, Woodside JV, Elborn JS, Young IS, McKinley MC (2011). Biomarkers of fruit and vegetable intake in human intervention studies:a systematic review. Crit Rev Food Sci Nutr.

[12] Barry TN, Allsop TF, Redekopp C (1986). The role of con-densed tannins in the nutritional value of Lotus pedunculatus for sheep. 5. Effects on the endocrine system and on adipose tissue metabolism. Br J Nutr.

[13] Basta G, Schmidt AM, De Caterina R (2004). Advanced glycation end products and vascular inflammation: implications for accelerated atherosclerosis in diabetes. Cardiovasc Res.

[14] Baxter GF, Sumeray MS, Walker JM (1996). Infarct size and magnesium:insights into LIMIT-2 and ISIS-4 from experimental studies. Lancet.

[15] Bei W, Peng W, Ma Y, Xu A (2005). Flavonoids from the leaves of Diospyros kaki reduce hydrogen peroxide-induced injury of NG108-15 cells. Life Sci.

[16] Bibi N, Khattak AB, Mehmood Z (2007). Quality improvement and shelf life extension of persimmon fruit Diospyros kaki. J Food Eng.

[17] Bravo L, Saura-Calixto F, Goni I (1992). Effects of dietary fibre and tannins from apple pulp on the composition of feces in rats. Br J Nutr.

[18] Bubba MD, Giordani E, Pippucci L, Cincinelli A, Checchini L, Galvan P (2009). Changes in tannins ascorbic acid and sugar contents in astringent persimmons during on-tree growth and ripening and in response to different postharvest treatments. J Food Comp Anal.

[19] Butt MS, Nazir A, Sultan MT, Schroën K (2008). Morus alba L Nature’s functional tonic. Trends Food Sci Technol.

[20] Chen G, Wang ZQ, Jia JM (2009). Three minor novel triterpenoids from the leaves of Diospyros kaki. Chem Pharm Bull (Tokyo).

[21] Chen K, Thomas SR, Keaney JF (2003). Beyond LDL oxidation: ROS in vascular signal transduction. Free Radic Biol Med.

[22] Chen KS, Zheng JT, Zhang SL, Gavin SR (1999). The role of ethylene in fruit ripening and softening. J Zhejiang Agr Univ.

[23] Chen XN, Fan JF, Yue X, Wu XR, Li LT (2008). Radical scavenging activity and phenolic compounds in persimmon Diospyros kaki L cv Mopan. J Food Sci.

[24] Cho IH, Gong JH, Kang MK, Lee EJ, Park JH, Park SJ (2014). Astragalin inhibits airway eotaxin-1 induction and epithelial apoptosis through modulating oxidative stress-responsive MAPK signaling. BMC Pulm Med.

[25] Dauchet L, Amouyel P, Dallongeville J (2009). Fruits, vegetables and coronary heart disease. Nat Rev Cardiol.

[26] Dembitsky VM, Poovarodom S, Leontowicz H, Leontowicz M, Vearasilp S, Trakhtenberg S (2011). The multiple nutrition properties of some exotic fruits: Biological activity and active metabolites. Food Res Int.

[27] Deng H, Wen Q, Luo Y, Huang Y, Huang R (2012). Influence of different extracts from persimmon leaves on the antioxidant activity in diabetic mice. Zhong Nan Da Xue Xue Bao Yi Xue Ban.

[28] Dewanjee S, Das AK, Sahu R, Gangopadhyay M (2009). Antidiabetic activity of Diospyros peregrina fruit: effect on hyperglycemia hyperlipidemia and augmented oxidative stress in experimental type 2 diabetes. Food Chem Toxicol.

[29] Dillard CJ, German JB (2000). Phytochemicals: nutraceuticals and human health. J Sci Food Agric.

[30] Dixon AR, Xie DY, Sharma SB (2005). Proanthocyanidins - a final frontier in flavonoid research?. New Phytologist.

[31] Ercisli S, Akbulut M, Ozdemir O, Sengul M, Orhan E (2007). Phenolic and antioxidant diversity among persimmon (Diospyrus kaki L.) genotypes in Turkey. Int J Food Sci Nutr.

[32] Fennema OR (1977). Loss of vitamins in fresh and frozen foods. Food Technol.

[33] Fiedor J, Burda K (2014). Potential role of carotenoids as antioxidants in human health and disease. Nutrients.

[34] Gali HU, Perchellet EM, Klish DS, Johnson JM, Perchellet JP (1992). Hydrolyzable tannins:potent inhibitors of hydroperoxide production and tumor promotion in mouse skin treated with 12-O-tetradecanoylphobol-13-acetate in vivo. Int J Cancer.

[35] Gato N, Kadowaki A, Hashimoto N, Yokoyama S, Matsumoto K (2013). Persimmon fruit tannin-rich fiber reduces cholesterol levels in humans. Ann Nutr Metab.

[36] Gaziano JM, Hennekens CH (1993). 1993. The role of beta- carotene in the prevention of cardiovascular disease. Ann N Y Acad Sci.

[37] Gondo M, Tanaka N, Tanaka T, Shimomura K, Nakanishi F, Ishimaru KA (1999). Naphthalene glycoside fromcallus cultures of Diospyros kaki. Phytochemistry.

[38] Gorinstein S, Bartnikowska E, Kulasek G, Zemser M, Trakhtenberg S (1998). Dietary persimmon improves lipid metabolism in rats fed diets containing cholesterol. J Nutr.

[39] Gorinstein S, Kulasek GW, Bartnikowska E, Leontowicz M, Zemser M, Morawiec M (2000). The effects of diets, supplemented with either whole persimmon or phenol-free persimmon, on rats fed cholesterol. Food Chem.

[40] Gorinstein S, Zachwieja Z, Folta M, Barton H, Piotrowicz J, Zemser M (2001). Comparative content of dietary fiber total phenolics and minerals in persimmon and apples. J Agric Food Chem.

[41] Gorinstein S, Zemser M, Haruenkit R, Chuthakorn R, Grauer F, Martin-Belloso O (1999). Comparative content of total polyphenols and dietary fiber in tropical fruits and persimmon. J Nutr Biochem.

[42] Gorinstein S, Zemser M, Weitz M, Halevy S, Deutsch J, Tilis K (1994). Fluorometric analysis of phenolics in Persimmons. Biosci Biotechnol Biochem.

[43] Gu H, Li C, Xu Y, Hu W, Chen M, Wan Q (2008). Structural features and antioxidant activity of tannin from persimmon pulp. Food Res Int.

[44] Han J, Kang S, Choue R, Kim H, Leem K, Chung S (2002). Free radical scavenging effect of Diospyros kaki Laminaria japonica and Undaria pinnatifida. Fitoterapia.

[45] Hanasaki Y, Ogawa S, Fukui S (1994). The correlation between active oxygens scavenging and antioxidative effects of flavonoids. Free Radic Biol Med.

[46] Harima S, Nakano R, Yamauchi S, Kitano Y, Yamamoto Y, Inaba A (2003). Extending shelf-life of astringent persimmon Diospyros kaki Thunb fruit by 1-MCP. Postharvest Biol Technol.

[47] Hertog MG, Kromhout D, Aravanis C, Blackburn H, Buzina R, Fidanza F (1995). Flavonoid intake and long-term risk of coronary heart disease and cancer in the seven countries study. Arch Intern Med.

[48] Hibasami H, Achiwa Y, Fujikawa T, Komiya T (1996). Induction of programmed cell death (apoptosis) in human lymphoid leukemia cells by catechin compounds. Anticancer Res.

[49] Homnava A, Payne J, Koehler P, Eitenmiller R (1990). Provitamin a (alpha-carotene, beta-carotene and beta-cryptoxanthin) and ascorbic acid content of Japanese and American persimmons. J Food Qual.

[50] Igual M, Castello ML, Ortola MD, Andres A (2008). Influence of vacuum impregnation on respiration rate mechanical and optical properties of cut persimmon. J Food Eng.

[51] Ikegami A, Akagi T, Potter D, Yamada M, Sato A, Yonemori K (2009). Molecular identification of 1-Cys peroxiredoxin and anthocyanidin/flavonol 3-O-galactosyltransferase from proanthocyanidin-rich young fruits of persimmon Diospyros kaki Thunb. Planta.

[52] Ikegami A, Eguchi S, Kitajima A, Inoue K, Yonemori K (2007). Identification of genes involved in proanthocyanidin biosynthesis of persimmon (Diospyros kaki) fruit. Plant Sci.

[53] Innami S, Tabata K, Shimizu J, Kusunoki K, Ishida H, Matsuguma M (1998). Dried green leaf powders of Jew's mellow (Corchorus), persimmon (Diosphyros kaki) and sweet potato (Ipomoea batatas poir) lower hepatic cholesterol concentration and increase fecal bile acid excretion in rats fed a cholesterol-free diet. Plant Foods Hum Nutr.

[54] Itamura H, Zheng Q, Akaura K (2005). Industry and research trend of Japanese persimmon. Acta Horticult.

[55] Ittah, Y (1993). Sugar content changes in persimmon fruits (Diospyros kaki L.) during artificial ripening with CO2:a possible connection to deastringency mechanisms. Food Chem.

[56] Jang I, Jo EK, Bae MS, Lee HJ, Jeon GI, Park E (2010). Antioxidant and antigenotoxic activities of different parts of persimmon (Diospyros kaki cv. Fuyu) fruit. J Med Plants Res.

[57] Jo C, Son, JH, Shin MG, Byun, MW (2003). Irradiation effects on color and functional properties of persimmon (Diospyros kaki L. folium) leaf extract and licorice (Glycyrrhiza Uralensis Fischer) root extract during storage. Radiat Physics Chem.

[58] Jung SK, Kim K, Tae K, Kong G, Kim MK (2013). The effect of raw vegetable and fruit intake on thyroid cancer risk among women:a case-control study in South Korea. Br J Nutr.

[59] Jung ST, Park YS, Zachwieja Z, Folta M, Barton H, Piotrowicz J (2005). Some essential phytochemicals and the antioxidant potential in fresh and dried persimmon. Int J Food Sci Nutr.

[60] Jung UJ, Park YB, Kim SR, Choi MS (2012). Supplementation of persimmon leaf ameliorates hyperglycemia, dyslipidemia and hepatic fat accumulation in type 2 diabetic mice. PLoS One.

[61] Kapiszewska M, Soltys E, Visioli F, Cierniak A, Zajac G (2005). The protective ability of the Mediterranean plant extract against the oxidative DNA damage. The role of the radical oxygen species and the polyphenol content. J Physiol Pharmacol.

[62] Kawakami K, Aketa S, Nakanami M, Iizuka S, Hirayama M (2010). Major water-soluble polyphenols, proanthocyanidins, in leaves of persimmon (Diospyros kaki) and their alpha-amylase inhibitory activity. Biosci Biotechnol Biochem.

[63] Kawase M, Motohashi N, Satoh K, Sakagami H, Nakashima H, Tani S (2013). Biological activity of persimmon (Diospyros kaki) peel extracts. Phytother Res.

[64] Khanal P, Oh WK, Thuong PT, Cho SD, Choi HS (2010). 24-hydroxyursolic acid from the leaves of the Diospyros kaki (Persimmon) induces apoptosis by activation of AMP-activated protein kinase. Planta Med.

[65] Khokhar S, Magnusdottir SGM (2002). Total phenol, catechin, and caffeine contents of teas commonly consumed in the United Kingdom. J Agric Food Chem.

[66] Kiechl S, Willeit J, Egger G, Poewe W, Oberhollenzer F (1997). Body iron stores and the risk of carotid atherosclerosis:Prospective results from the Bruneck study. Circulation.

[67] Kim SH, Cho SS, Simkhada JR, Park SJ, Lee HJ, Kim TS (2010). Effects and action mechanism of Diospyros kaki on the differentiation of human leukemia HL-60 cells. Oncol Rep.

[68] Kim SY, Jeong SM, Kim SJ, Jeon KI, Park E, Park HR (2006). Effect of heat treatment on the antioxidative and antigenotoxic activity of extracts from persimmon (Diospyros kaki L.) peel. Biosci Biotechnol Biochem.

[69] Kim YJ, Yokozawa T (2009). Modulation of oxidative stress and melanogenesis by proanthocyanidins. Biol Pharm Bull.

[70] Kotani M, Matsumoto M, Fujita A, Higa S, Wang W, Suemura M (2000). Persimmon leaf extract and astragalin inhibit development of dermatitis and IgE elevation in NC/Nga mice. J Allergy Clin Immunol.

[71] Kromhout D, Menotti A, Kesteloot H, Sans S (2002). Prevention of coronary heart disease by diet and lifestyle:evidence from prospective cross-cultural, cohort, and intervention studies. Circulation.

[72] Kumazawa S, Taniguchi M, Suzuki Y, Shimura M, Kwon MS, Nakayama T (2002). Antioxidant activity of polyphenols in carob pods. J Agric Food Chem.

[73] Lapshina EA, Sudnikovich EJ, Maksimchik JZ, Zabrodskaya SV, Zavodnik LB, Kubyshin VL (2006). Antioxidative enzyme and glutathione S-transferase activities in diabetic rats exposed to long-term ASA treatment. Life Sci.

[74] Laranjinha JA, Almeida LM, Maderia VM (1994). Reactivity of dietary phenolic acids with peroxyl radicals:antioxidant activity upon low-density lipoprotein peroxidation. Biochem Pharmacol.

[75] Larsson M, Vorrsjö E, Talmud P, Lookene A, Olivecrona G (2013). Apolipoproteins C-I and C-III inhibit lipoprotein lipase activity by displacement of the enzyme from lipid droplets. J Biol Chem.

[76] Lee JH, Lee YB, Seo WD, Kang ST, Lim JW, Cho KM (2012). Comparative studies of antioxidant activities and nutritional constituents of persimmon juice (Diospyros kaki L. cv. Gapjubaekmok). Prev Nutr Food Sci.

[77] Lee JS, Lee MK, Ha TY, Bok SH, Park HM, Jeong KS (2006). Supplementation of whole persimmon leaf improves lipid profiles and suppresses body weight gain in rats fed high-fat diet. Food Chem Toxicol.

[78] Lee YA, Cho EJ, Tanaka T, Yokozawa T (2007). Inhibitory activities of proanthocyanidins from persimmon against oxidative stress and digestive enzymes related to diabetes. J Nutr Sci Vitaminol (Tokyo).

[79] Lee YA, Cho EJ, Yokozawa T (2008). Effects of proanthocyanidin preparations on hyperlipidemia and other biomarkers in mouse model of type 2 diabetes. J Agric Food Chem.

[80] Lee YA, Cho EJ, Yokozawa T (2010). Oligomeric proanthocyanidins improve memory and enhance phosphorylation of vascular endothelial growth factor receptor-2 in senescence-accelerated mouse prone/8. Br J Nutr.

[81] Lee YA, Kim YJ, Cho EJ, Yokozawa T (2007). Ameliorative effects of proanthocyanidin on oxidative stress and inflammation in streptozotocin-induced diabetic rats. J Agric Food Chem.

[82] Luo Z (2007). Effect of 1-methylcyclopropene on ripening of postharvest persimmon (Diospyros kaki L.) fruit. LWT Food Sci Technol.

[83] Manach C, Scalbert A, Morand C, Rémésy C, Jiménez L (2004). Polyphenols: food sources and bioavailability. Am J Clin Nutr.

[84] Mashima R, Witting PK, Stocker R (2001). Oxidants and antioxidants in atherosclerosis. Curr Opin Lipidol.

[85] Mathews-Roth MM (1993). Carotenoids in erythropoietic pro-toporphyria and other photosensitivity diseases. Ann N Y Acad Sci.

[86] Matsumoto K, Watanabe Y, Ohya MA, Yokoyama S (2006). Young persimmon fruits prevent the rise in plasma lipids in a diet-induced murine obesity model. Biol Pharm Bull.

[87] Matsumoto K, Yokoyama S, Gato N (2010). Bile acid-binding activity of young persimmon (Diospyros kaki) fruit and its hypolipidemic effect in mice. Phytother Res.

[88] Matsumoto K, Yokoyama S, Gato N (2008). Hypolipidemic effect of young persimmon fruit in C57BL/6.KOR-ApoEshl mice. Biosci Biotech Biochem.

[89] Matsuo T, Itoo S (1978). The chemical structure of kaki-tannin from immature fruit of the persimmon (Diospyros kaki L.). Agric Biol Chem.

[90] Matsuura E, Kobayashi K, Tabuchi M, Lopez LR (2006). Oxidative modification of low-density lipoprotein and immune regulation of atherosclerosis. Prog Lipid Res.

[91] Michel TM, Pulschen D, Thome J (2012). The role of oxidative stress in depressive disorders. Curr Pharm Des.

[92] Miller PE, Snyder DC (2012). Phytochemicals and cancer risk: a review of the epidemiological evidence. Nutr Clin Pract.

[93] Moon YJ, Cha YS (2008). Effects of persimmon-vinegar on lipid metabolism and alcohol clearance in chronic alcohol-fed rats. J Med Food.

[94] Mure K, Takeshita T, Morioka I, Arita M (2007). Effects of kakisu (persimmon vinegar) on plasma antioxidant power and urinary 8-isoprostane level. Nihon Eiseigaku Zasshi.

[95] Nakano R, Inoue S, Kubo Y, Inaba A (2002). Water stress induced ethylene in the calix triggers autocatalytic ethylene production and fruit softening in ‘Tonewase’ persimmon grown in a heated plastic-house. Postharvest Biol Technol.

[96] Ohguchi K, Nakajima C, Oyama M, Iinuma M, Itoh T, Akao Y (2010). Inhibitory effects of flavonoid glycosides isolated from the peel of Japanese persimmon (Diospyros kaki 'Fuyu') on melanin biosynthesis. Biol Pharm Bull.

[97] Ouyang P, Bei W, Lai W, Peng W (2004). Effects of flavone from leaves of Diospyros kaki on rat vascular smooth muscle cells proliferation stimulated by native low-density lipoprotein in vitro. Zhong Yao Cai.

[98] Ozen A, Colak A, Dincer B, Guner S (2004). A diphenolase from persimmon fruits (Diospyros kaki L, Ebenaceae). Food Chem.

[99] Park SY, Park SH, Jeon SM, Park YB, Lee SJ, Jeong TS (2002). Effect of rutin and tanic acid supplements on cholesterol metabolism in rats. Nutr Res.

[100] Park YS, Jung ST, Kang SG, Delgado-Licon E, Ayala ALM, Tapia MS (2006). Drying of persimmons (Diospyros kaki L.) and the following changes in the studied bioactive compounds and the total radical scavenging activities. LWT-Food Sci Technol.

[101] Paul M, Mehr, AP, Kreutz R (2006). Physiology of local renin-angiotensin systems. Physiol Rev.

[102] Piretti MV (1991). Polyphenol constituents of the Diospyros kaki fruit. A review. Fitoterapia.

[103] Plumb GW, De Pascual-Teresa S, Santos-Buelga C, Cheynier V, Williamson G (1998). Antioxidant properties of catechins and proanthocyanidins:effect of polymerisation, galloylation and glycosylation. Free Radic Res.

[104] Prakash P, Krinsky NI, Russell RM (2000). Retinoids, carotenoids and human breast cancer cell cultures:A review of differential effects. Nutr Rev.

[105] Rahman MA, Islam AKMS, Khair A, Bala BK (2002). Effect of non polar gases on the storage of persimmon fruits at different temperatures. Pak J Biol Sci.

[106] Ramasubbu N, Ragunath C, Mishra PJ, Thomas LM, Gyémánt G, Kandra L (2004). Human salivary alpha-amylase Trp58 situated at subsite -2 is critical for enzyme activity. Eur J Biochem.

[107] Remuzzi G, Ruggenenti P (2006). Overview of randomised trials of ACE inhibitors. Lancet.

[108] Rice-Evans CA, Miller NJ, Paganga J (1997). Antioxidant properties of phenolic compounds. Trends in Plant Sci.

[109] Robertson RP, Harmon J, Tran PO, Tanaka Y, Takahashi H (2003). Glucose toxicity in beta-cells:type 2 diabetes, good radicals gone bad, and the glutathione connection. Diabetes.

[110] Roller M, Clune Y, Collins K, Rechkemmer G, Watzl B (2007). Consumption of prebiotic inulin enriched with oligofructose in combination with the probiotics Lactobacillus rhamnosus and Bifidobacterium lactis has minor effects on selected immune parameters in polypectomised and colon cancer patients. Br J Nutr.

[111] Ros E, Hu FB (2013). Consumption of plant seeds and cardiovascular health:epidemiological and clinical trial evidence. Circulation.

[112] Sa YS, Kim SJ, Choi HS (2005). The anticoagulant fraction from the leaves of Diospyros kaki L. has an antithrombotic activity. Arch Pharm Res.

[113] Sakanaka S, Tachibana Y, Okada Y (2005). Preparation and antioxidant properties of extracts of Japanese persimmon leaf tea (kakinoha-cha). Food Chem.

[114] Sarkar A, Bishayee A, Chatterjee M (1995). Beta-carotene prevents lipid peroxidation and red blood cell membrane protein damage in experimental hepatocarcinogenesis. Cancer Biochem Biophys.

[115] Singh BB, Vinjamury SP, Der-Martirosian C, Kubik E, Mishra LC, Shepard NP (2007). Ayurvedic and collateral herbal treatments for hyperlipidemia:a systematic review of randomized controlled trials and quasi-experimental designs. Altern Ther Health Med.

[116] Sultan MT, Butt MS, Anjum FM, Jamil A (2009). Influence of black cumin fixed and essential oil supplementation on markers of myocardial necrosis in normal and diabetic rats. Pak J Nutr.

[117] Sun L, Zhang J, Lu X, Zhang L, Zhang Y (2011). Evaluation to the antioxidant activity of total flavonoids extract from persimmon (Diospyros kaki L.) leaves. Food Chem Toxicol.

[118] Suzuki T, Someya S, Hu F, Tanokura M (2005). Comparative study of catechin compositions in five Japanese persimmons (Diospyros kaki). Food Chem.

[119] Takahashi M, Watanabe H, Kikkawa J, Ota M, Watanabe M, Sato Y (2006). Carotenoids extraction from Japanese persimmon (Hachiya-kaki) peels by supercritical CO2 with ethanol. Anal Sci.

[120] Veberic R, Jurhar J, Mikulic-Petkovsek M, Stampar F, Schmitzer V (2010). Comparative study of primary and secondary metabolites in 11 cultivars of persimmon fruit (Diospyros kaki L.). Food Chem.

[121] Wang H, Leng P, Zhao G, Ji Q (2004). Advances in research of storage technology for persimmon. J Fruit Sci.

[122] Wild S, Roglic G, Green A, Sicree R, King H (2004). Global prevalence of diabetes. Diabetes Care.

[123] Woodside JV, Young IS, McKinley MC (2013). Fruit and vegetable intake and risk of cardiovascular disease. Proc Nutr Soc.

[124] Wu P-W, Hwang LS (2002). Determination of soluble persimmon tannin by high performance gel permeation chromatography. Food Res Int.

[125] Xiao J, Ni X, Kai G, Chen X (2013). A review on structure-activity relationship of dietary polyphenols inhibiting α-amylase. Crit Rev Food Sci Nutr.

[126] Xing N, Chen Y, Mitchell SH, Young CYF (2001). Quercetin inhibits the expression and function of the androgen receptor in LNCaP prostate cancer cells. Carcinogenesis.

[127] Yamada M, Tairab S, Ohtsuki M, Sato A, Iwanamia H, Yakushijia H (2002). Varietal differences in the ease of astringency removal by carbon dioxide gas and ethanol vapor treatments among Oriental astringent persimmons of Japanese and Chinese origin. Sci Horticulturae.

[128] Yokozawa T, Kim YA, Kim HY, Lee YA, Nonaka G (2007). Protective effect of persimmon peels polyphenol against high glucose-induced oxidative stress in LLC-PK1 cells. Food Chem Toxicol.

[129] Yokozawa T, Park CH, Noh JS, Roh SS (2014). Role of oligomeric proanthocyanidins derived from an extract of persimmon fruits in the oxidative stress-related aging process. Molecules.

[130] Yonemitsu S, Nishimura H, Shintani M, Inoue R, Yamamoto Y, Masuzaki H (2001). Troglitazone induces GLUT4 translocation in L6 myotubes. Diabetes.

[131] Zhao M, Yang B, Wang J, Liu Y, Yu L, Jiang Y (2007). Immunomodulatory and anticancer activities of flavonoids extracted from litchi (Litchi chinensis Sonn) pericarp. Int Immunopharmacol.

[132] Zheng GH, Sugiura A (1990). Changes in sugar composition in relation to invertase activity in the growth and ripening of persimmon (Diospyros kaki) fruits. J Jap Soc Horticul Sci.

[133] Zheng QL, Nakatsuka A, Itamura H (2006). Involvement of negative feedback regulation in wound-induced ethylene synthesis in ‘Saijo’ persimmon. J Agric Food Chem.

[134] Zheng, QL, Nakatsuka A, Taira S, Itamura H (2005). Enzymatic activities and gene expression of 1-aminocyclopropane-1-carboxylic acid (ACC) synthase and ACC oxidase in persimmon fruit. Postharvest Biol Technol.

[135] Zhou C, Sheng Y, Zhao D, Wang Z, Tao J (2010). Variation of oleanolic and ursolic acid in the flesh of persimmon fruit among different cultivars. Molecules.

[136] Ziel FH, Venkatesan N, Davidson MB (1988). Glucose transport is rate limiting for skeletal muscle glucose metabolism in normal and STZ-induced diabetic rats. Diabetes.

[137] Zou B, Li CM, Chen JY, Dong XQ, Zhang Y, Du J (2012). High molecular weight persimmon tannin is a potent hypolipidemic in high-cholesterol diet fed rats. Food Res Int.

